# Preliminary Phytochemical Analysis and Evaluation of the Biological Activity of *Leonotis nepetifolia* (L.) R. Br Transformed Roots Extracts Obtained through *Rhizobium rhizogenes*-Mediated Transformation

**DOI:** 10.3390/cells10051242

**Published:** 2021-05-18

**Authors:** Tomasz Kowalczyk, Anna Merecz-Sadowska, Patricia Rijo, Vera M. S. Isca, Laurent Picot, Marzena Wielanek, Tomasz Śliwiński, Przemysław Sitarek

**Affiliations:** 1 Department of Molecular Biotechnology and Genetics, University of Lodz, 90-237 Lodz, Poland; tomasz.kowalczyk@biol.uni.lodz.pl; 2 Department of Computer Science in Economics, University of Lodz, 90-214 Lodz, Poland; anna.merecz-sadowska@uni.lodz.pl; 3 Center for Research in Biosciences & Health Technologies (CBIOS), Universidade Lusófona de Humanidades e Tecnologias, 1740-024 Lisboa, Portugal; p1609@ulusofona.pt (P.R.); vera.isca@ulusofona.pt (V.M.S.I.); 4 Instituto de Investigação do Medicamento (iMed.ULisboa), Faculty of Pharmacy, Universidade de Lisboa, 1649-003 Lisboa, Portugal; 5 Littoral Environnement et Sociétés LIENSs, La Rochelle Université, UMRi CNRS 7266 LIENSs, 17042 La Rochelle, France; laurent.picot@univ-lr.fr; 6 Department of Plant Physiology and Biochemistry, Faculty of Biology and Environmental Protection, University of Lodz, Banacha 12/16, 90-237 Lodz, Poland; marzena.wielanek@biol.uni.lodz.pl; 7 Laboratory of Medical Genetics, Faculty of Biology and Environmental Protection, University of Lodz, 90-236 Lodz, Poland; 8 Department of Biology and Pharmaceutical Botany, Medical University of Lodz, 90-151 Lodz, Poland

**Keywords:** *Leonotis nepetifolia*, hairy roots, *Rhizobium rhizogenes* transformation, cytotoxic activity, ROS levels, DNA repair and protective effect

## Abstract

According to the present knowledge, this is the first report on establishing transformed root cultures of *Leonotis nepetifolia* after *Rhizobium rhizogenes*-mediated transformation. The preliminary phytochemical analysis showed differences in the content of phenols and flavonoids in transformed and nontransformed roots. The dominant compounds in the analyzed extracts were (+)-catechin (5464 and 6808 µg/g DW), *p*-coumaric acid (2549 and 4907 µg/g DW), *m*-coumaric acid (1508 and 2048 µg/g DW) and rosmarinic acid (1844 and 2643 µg/g DW) for nontransformed (LNNR) and transformed (LNTR4) roots, respectively. Initial biological studies carried out on LNNR, and LNTR4 extracts showed a cytotoxic effect on the A549 lung, HCC1937 breast and leukemia NALM-6 cell lines, antioxidants, as well as repair and protection against DNA damage induced by H_2_O_2_ in HUVEC cells. Due to the stronger effect of the LNTR4 root extract, which can be a relatively efficient and cheap source of bioactive secondary metabolites, further biological analyses are needed to discover in detail their potentially valuable biological properties.

## 1. Introduction

Plant tissue cultures are used in biotechnology as a potential source for the production of useful secondary metabolites. Thanks to this technique, it is often possible to increase the synthesis and accumulation of valuable secondary metabolites and to create optimal conditions for growth and development, independent of geographic and climatic factors and without harmful interference to the natural environment [1,2]. Hairy roots are currently an extremely popular model among plant tissue cultures due to their unlimited and rapid growth compared to nontransformed roots, genetic stability, and the ability to synthesize a wide spectrum of biologically active compounds. For these reasons, they can be used to produce commercially valuable products even on a large scale [3]. It is also known that the hairy roots of some plant species can produce completely new secondary metabolites, which are not found in nontransformed plants and, therefore, offer the possibility of discovering entirely new biological properties. Plant genetic transformation using the natural vector system like *Rhizobium rhizogenes* (previously referred to as *Agrobacterium rhizogenes*) is very often used nowadays to genetically modify the plant genome [4,5]. It has been demonstrated that the root-inducing plasmid present in *R. rhizogenes* is involved in the transformation mechanism, and its region (T-DNA) is integrated into the genome of plant cells. As a consequence, hairy root disease develops, causing practically unlimited root proliferation at the infection site [2]. There are many reports that have successfully demonstrated the obtaining of hairy roots and the increase in the production of secondary metabolites [6,7,8,9]. Our earlier studies showed a positive transformation effect and produced different clones of *Leonurus sibiricus* L. hairy roots with increased production of phenolic acids [10,11].

The plant *Leonotis nepetifolia* (L.) R. Br. belongs to the family Lamiaceae, is a robust annual herb, about 1–2 m tall, its inflorescence globose is whorled at upper nodes with orange flowers, and it is also known under the name of klip dagga, lion’s ear or cordão-defrade [12,13]. It is widespread throughout West India, South America, and the African continent. *Leonotis nepetifolia* is an important medicinal plant with a long history of numerous traditional medicinal uses in various countries. It is used for cough, fever, stomachache, skin infection, rheumatism, dysmenorrhea, and kidney dysfunction [12,14]. In particular, it has a reputation in Indian traditional medical systems, such as Ayurveda, Unani, and Siddha [13]. Roots of this plant have been used in *Brachat, Guduchi Taila* and *Mritsanjivani Sura* in Ayurvedic formulations and applied in indications of *swasa* (asthma and bronchitis) *kandu* (fever), and *visa* (poisonous conditions). Leaves are used in diabetes in Trinidad and Tobago and in asthma and cough in Africa. Seeds are a remedy for burns in India. The whole plant, in turn, is used for menstrual pain and unspecified female complaints [12,13,15]. Currently, numerous studies on this species have demonstrated many biological activities, including cytotoxic, anti-inflammatory, antispasmodic, antibacterial, antifungal, antioxidant, antiemetic, antidiabetic, analgesic and antidiarrheal [13,16,17,18,19]. Phytochemical analysis of this species revealed the presence of volatile oils, carbohydrates, terpenoids, saponins, flavonoids, along with phenolic acids, proteins, amino acids, phytosterols, alkaloids and fixed oils [20,21,22].

In this work, we reported for the first time the successful transformation, establishment of in vitro transformed roots and preliminary phytochemical analysis of *Leonotis nepetifolia* nontransformed and transformed root extracts. We assessed the cytotoxic and antioxidant activity. In addition, a repair and protection effect on DNA in HUVEC cells induced by H_2_O_2_ was presented.

## 2. Materials and Methods

### 2.1. Leonotis nepetifolia Seeds Surface Sterilization Protocol

**Figure 1:**
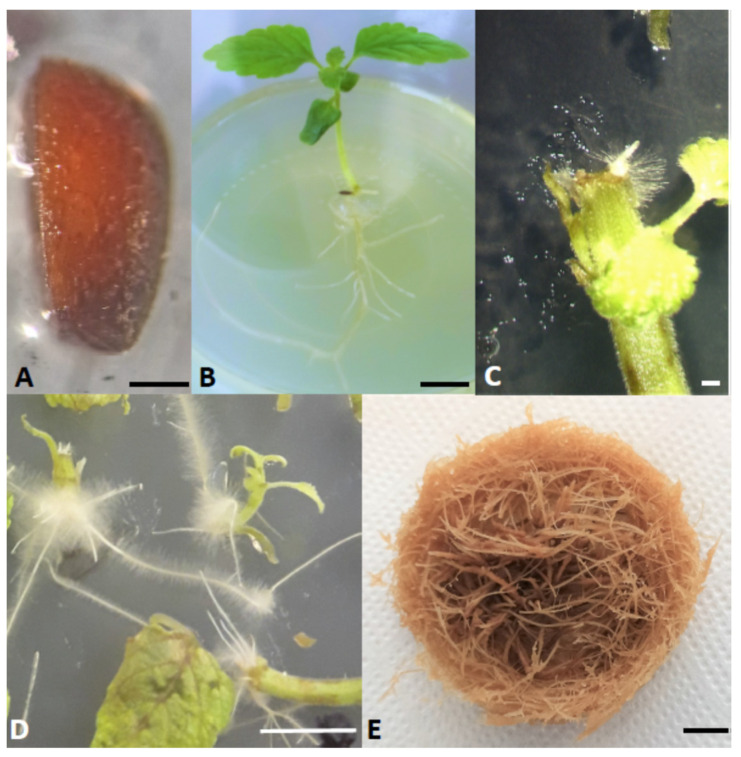
(**A**) Sterile seed on solid medium after the surface sterilization procedure, (**B**) 14 day-old seedling grown in vitro used for *Rhizobium*-mediated transformation, (**C**) first hairy roots appearing on plant material 14 days after transformation, (**D**) hairy roots on plant tissue 21 days after transformation, (**E**) hairy roots grown in flasks in liquid medium. Bar (**A**,**C**) = 1 mm, (**B**,**D**,**E**) = 1 cm.

*Leonotis nepetifolia* seeds were obtained from the Botanical Garden of the Technical University Braunschweig, Germany. The botanical identity of plants was confirmed by Przemysław Sitarek according to the International Plant Name Index (IPNI) using http:/powo.science.kew.org/ (accessed on 6 April 2021). A voucher specimen was deposited at the Department of Biology and Pharmaceutical Botany, Medical University of Lodz, Poland. The surface sterilization of seeds was carried out as follows: about 200 seeds were pre-rinsed with 200 mL of distilled sterile water. The seeds were then rinsed in 70% ethanol for 1 min. After this, the seeds were rinsed several times with plenty of sterile water. Then, a 4% sodium hypochlorite solution with the addition of Tween-20 drop was added to the seeds to increase the contact of the surface of the seed with the disinfectant, and they were rinsed for 10 min with gentle agitation. After this time, the seeds were rinsed 5 times with 100 mL of sterile water. The seeds were incubated in water for 10 min on the final rinse. The seeds sterilized in this way were dried on sterile sheets of filter paper and then placed in Petri dishes containing agar solidified 1/2 Murashige and Skoog medium without sucrose (Figure 1A). The seeds were kept at 23 °C. Young 5–6 cm seedlings were transferred to 0.8% agar solidified Murashige and Skoog medium containing 3% sucrose.

### 2.2. Leonotis nepetifolia Seedlings Inoculation with Rhizobium rhizogenes

*Rhizobium rhizogenes* strain A4 was used to infect plant material. Bacteria from a single colony growing on a Petri dish with solid YEP medium (10 g/L yeast extract, 10 g/L Bacto peptone and 5 g/L NaCl, 1.5% agar, pH = 7.0) were used to start shaken bacterial culture. 5 mL of liquid YEP medium was inoculated with bacteria and cultured at 28 °C with vigorous shaking (180 rpm) to an optical density OD600 = 0.5. Then the bacteria were centrifuged at 4000 rpm, the supernatant was removed, and the bacterial pellet was suspended in the liquid MS medium. The bacterial suspension prepared in this way was used to transform the plant material. The starting material for transformation was 14 day-old seedlings grown in vitro (Figure 1B). In this work, 2 procedures of genetic transformation of a plant were used. In the first method (dipping method, DM), young seedlings were cut off the roots, and the cut site was inoculated with bacteria by immersing it to a depth of 1–2 mm in the *R. rhizogenes* suspension. Then the excess bacteria was removed onto sterile filter paper, and the seedlings were placed upside down in Petri dishes on a solid MS medium (0.8% agar). In the second method, bacterial suspension was injected into the hypocotyl using an insulin syringe (injection method, IM). Each method was performed both in the absence or presence of 100 µM acetosyringone. All seedlings were incubated in the dark at 25 °C for 4 days. Then they were washed in cefotaxime solution (500 mg/L), dried on sterile filter paper and transferred into MS solid medium with 3% sucrose. The seedlings after transformation were grown at 25 °C until the appearance of hairy roots at 16 h light and 8 h dark cycles. The 2–3 cm hairy roots were cut from plant explants and transferred to MS solid medium with 3% sucrose and 250 mg/L cefotaxime (to eliminate the *Rhizobium*) and grown in the dark at 25 °C. Roots were transferred to a new medium every 2 weeks. After 4 passages, the roots were transferred to 50 mL of liquid MS medium with 3% sucrose and 250 mg/L cefotaxime and grown in the dark at 25 °C with vigorous shaking (150 rpm). Hairy roots were passaged every 3 weeks on a new medium to eliminate all bacteria. After the 4th passage, the concentration of the antibiotic was gradually reduced (125 mg/L, 62.5 mg/L) until it was finally fully eliminated. In this work, 7 clones (LNTR1-7) of transformed roots were finally obtained. One transformed LNTR4 root line (Figure 1E) was selected for further study, which showed the fastest growth in the liquid medium and produced more lateral roots than the other seven lines. The cultures were kept at 25 °C in the dark on a rotary shaker at 80 rpm. Subculture was performed every 5 weeks, transferring approximately 0.5 g of fresh root biomass to fresh medium. The untransformed roots were grown under the same conditions as transformed roots without using growth regulators. These roots were passaged onto a new medium every 21 days.

### 2.3. DNA Isolation and Polymerase Chain Reaction

Hairy roots induced on the plant tissue were analyzed for the presence of *rol* genes. The isolation of genomic DNA was performed using the Genomic mini AX plant kit (A&A Biotechnology, Gdansk, Poland) according to the manufacturer’s instructions. 100 mg of the fresh root was used for isolation. PCR was performed using a set of a specific primer for the *rol*B and *rol*C genes. The *rol* B gene fragment was amplified using the following primers: - 5′-GCTCTTGCAGTGCTAGATTT-3′ and - 5′-GAAGGTGCAAGCTACCTCTC-3′, while for the *rol*C gene - 5′-GAAGACGACCTGTGTTCTC-3′ i - 5′-CGTTCAAACGTTAGCCGATT-3′ were used. The PCR reaction was performed in 20 µL with 100 ng of DNA from normal and hairy roots as a template. A PCR Mix kit (A&A Biotechnology, Gdansk, Poland) was used for the reaction. PCR for both genes was performed as follows: initial denaturation at 95 °C for 5 min, followed by 35 cycles: 1 min denaturation at 95 °C, 1 min annealing in 49 °C (for *rol*B) or 52 °C (for *rol*C), and 1 min extension in 72 °C with a final extension of 72 °C for 5 min using Biometra Uno II thermocycler (Göttingen, Germany). The PCR products were analyzed by electrophoretic separation on a 1.5% ethidium bromide-stained agarose gel. The obtained results were documented using a transilluminator (Vilber Lourmat, Marine la Valeé, France) equipped with a digital gel documentation system.

### 2.4. Plant Materials and Extract Preparation from LNNR and LNTR4 Roots of L. nepetifolia

In this study, two different extracts from normal (LNTR) and transformed (LNTR4) roots of *Lenonotis nepetifolia* were used. Briefly, the lyophilized and powdered plant materials were extracted for 15 min with 80% (*v/v*) aqueous methanol (500 mL) at 35 °C using an ultrasonic bath and then twice with 300 mL of the same solvent for 15 min. Next, both extracts were filtered, combined, and evaporated under reduced pressure and then were lyophilized according to our previous studies [10].

### 2.5. Phytochemical Analysis

Chromatographic analysis of the tested extracts was carried out using an HPLC system (Dionex, Sunnyvale, CA, USA) equipped with a photodiode array and fluorometric detectors. Separation of the compounds was achieved on an RP column (aQ Hypersil GOLD; 250 × 4.6 mm, 5 μm) linked to a guard column (GOLD aQ Drop-In Guard; 10 × 4 mm, 5 μm; Polygen, Poland) at 25 °C using a mobile phase composed of (a) water and (b) methanol, both with 0.1% formic acid. Quantification was based on the calibration curves for reference standards prepared using absorbance or emission wavelength optimal for phenolic compounds (A235, 280, 325, 375 nm, Em 420 nm). LC-MS/MS was carried out using API LC/MS/MS system (Applera, Foster City, CA, USA) with electrospray ionization (ESI) source equipped with Dionex (Sunnyvale, CA, USA) HPLC system. Separation was achieved on a Q Hypersil GOLD column (C18, 2.1 × 150 mm, 5 μm) at 30 °C using a mobile phase as described above for HPLC and a flow rate of 0.2 mL/min. LC–MS/MS and HPLC analyses were performed as described previously according to Sitarek et al. [23].

### 2.6. Cell Culture

The studies were conducted on human lung adenocarcinoma A549 (CCL-185; ATCC, Manassas, VA, USA) cell line, breast cancer cell line HCC1937 (CRL-2336, ATCC, Manassas, VA, USA) and NALM-6 ALL cells (CRL-3273, ATCC, Manassas, VA, USA). The cell lines were maintained in DMEM media (ThermoFisher Scientific, Waltham, MA, USA) (for A549) or RPMI 1640 media (HTC1937 and NALM6) supplemented with 10% (*v/v*) HI fetal bovine serum (FBS) and 100 U/mL penicillin, 100 μg/mL streptomycin at 37 °C in 5% CO_2_ atmosphere. Cell culture media and supplements were purchased from Lonza (Basel, Switzerland). In addition, human umbilical vein endothelial cells (HUVEC) were also used for the study. These cells were purchased from Gibco (Cascade Biologics, Portland, OR, USA) catalog number C0035C) and cultured in medium 200 (Gibco, catalog number M-200–500), supplemented with low serum G growth supplement kit (LSGS Kit; Gibco, catalog number S003K) at 37 °C and 5% CO_2_ in an incubator (Galaxy^®^ R-CO2 Incubator, New Brunswick Scientific, New Brunswick, NJ, USA) in a humidified atmosphere.

### 2.7. Determination of Cytotoxic Effect of LNNR and LNTR4 Root Extracts by MTT Assay

Cell viability was examined by the ability of the cells to cleave the tetrazolium salt MTT (3-(4,5 dimethylthiazol-2-yl)-2,5-diphenyl tetrazolium bromide) by the mitochondrial enzyme succinate dehydrogenase following the procedure as described earlier. Briefly, the cells were seeded in 96-well plates at a density of 2.5 × 10^4^ to 3.5 × 10^4^ cells/well. Following 24 h incubation and attachment (or right after seeding NALM-6), cells were treated with different concentrations of LNNR and LNTR4 root extracts for 24 h. Following that, the MTT compound was added to the culture media, and the cells were placed in the incubator for 2–4 h, after which media containing MTT was discarded, and formazan crystals were dissolved by adding DMSO. Absorbance measurement was performed at 595 nm using the GloMax system from Promega [10,24].

### 2.8. Measurement of Cytoplasmic ROS Levels

The level of ROS accumulation after induction of oxidative stress with 100 µM of H_2_O_2_ was measured using the redox-sensitive fluorescent dye-DCFH-DA. The cells were incubated with 100 µM of H_2_O_2_ alone or combined with analyzed extracts in the highest tested concentrations (100 µM). After 24 h cells were washed, DCFH-DA at the final concentration of 5 μM (prepared in Tyrode’s Ca^2^+/Mg^2^+-free-buffer) was added, and the cells were incubated at 37 °C for 45 min. Fluorescence was measured (excitation 480 nm, emission 510 nm) using GloMax device (Promega, Madison, WA, USA), and the results were expressed as a percentage of the control (untreated cells).

### 2.9. 2,2-Diphenyl-1-Picrylhydrazyl (DPPH) Assay

The antioxidant activity of LNNR and LNTR4 root extracts was measured by the DPPH method, as described by Rijo et al. [25]. 10 μL of each sample were added to a 990 μL solution of DPPH (0.002% in methanol). The mixture was incubated for 30 min at room temperature. The absorbance was measured at 517 nm in UV-vis spectrophotometer *U-*2010 Hitachi. The absorbance was corrected against a corresponding blank, and the antioxidant activity was calculated using the following equation:

(1) $$AA\left(\%\right)=\frac{ADPPH-ASample}{ADPPH}\times 100$$

where *AA* is the antioxidant activity, *ADPPH* is the absorption of *DPPH* against the blank, and *A* sample is the absorption of the extractor control against the blank. Tests were carried out in triplicate at a sample concentration at 10 mg of dry LNNR and LNTR4 root extracts/mL. Quercetin was the reference standard used in this procedure, under the same conditions as the samples [26].

### 2.10. DNA Repair and Protective Effect on H_2_O_2_-Induced HUVEC Cells after Treatment of LNNR and LNTR4 Root Extracts of L. nepetifolia Assessment by Comet Assay Method

The DNA damage and repair were evaluated using a single-cell gel electrophoresis assay under alkaline (pH > 13) conditions. The technique was conducted according to Singh et al. with slight modifications [27]. For assessing the DNA damage and repair efficiency after treatment with hydrogen peroxide, the experiment was conducted as follows: The negative control was HUVEC cells (2 × 10^5^ per mL) incubated without LNNR and LNTR4 root extracts and damaging agents. The positive control constituted cells incubated with 75 µM of hydrogen peroxide (H_2_O_2_) in Eppendorf tubes for 10 min at 4 °C and subjected to repair incubation in the medium during 0, 15, 30, 60 and 120 min. The other samples were centrifuged after exposure to the damage compounds. The medium was removed, and the cells were washed with PBS. Samples were suspended in medium and plant extract (LNNR and LNTR4) to give the final concentration of 50, 150, and 250 µM. Those cells were subjected to 0, 15, 30, 60, and 120 min of repaired incubation at 37 °C in a growth medium. For assessing the protective plant extract impact, the experiment was conducted as follows: The cells suspension (2 × 10^5^ per mL) was pre-incubated in a medium with plant extract (LNNR and LNTR4) to give the final concentration 50, 150 and 250 µM for 24 h at 37 °C. After then, cells were centrifuged (1400 rpm for 10 min at 4 °C), suspended in medium, and treated with 75 µM of H_2_O_2_ in Eppendorf tubes for 10 min at 4 °C. The negative control was HUVEC cells incubated without plant extracts and damaging agents. The positive control constituted cells treated with 75 µM of H_2_O_2_ in Eppendorf tubes for 10 min at 4 °C. After incubation, all samples were then centrifuged, suspended in 0.75% agarose with low gelling temperature, spread onto microscope slides. The slides were prior precoated with 0.5% agarose with normal gelling temperature and covered with slide coverslips. The microscope glasses were put into ice, and after the agarose gel solidified, the coverslips were removed. Then the slides were incubated in lysis buffer (2.5 M NaCl, 100 mM EDTA, 10 mM Tris adjusted to pH 10 and supplemented with 1% Triton X-100 before use) at 4 °C for a minimum of one hour. Samples were then rinsed and incubated under alkaline conditions, pH > 13 in expanding buffer (300 mM NaOH, 1 mM EDTA) for 20 min and subjected to electrophoresis in the same buffer for 20 min at 0.7 V/cm and 30 mA. They were washed in water, drained, and stained with 2 µg/mL 4′,6-diamidino-2-phenylindole dihydrochloride (DAPI) and covered with coverslips. Further procedures were performed according to our previous study [23].

### 2.11. Assessment of General Toxicity—Brine Shrimp Lethality Bioassay

The general toxicity of each sample was assessed by the *Artemia salina* lethality bioassay, as described by Ntungwe et al. [26]. A 24-well plate was used, and samples of each LNNR and LNTR4 root extracts were prepared at a concentration of 500 µg/mL, with dilutions performed in the salt medium. Ranging numbers of 10 to 15 larvae were added to each well-containing salt medium. Afterward, both extracts were added to corresponding wells, and the plate was stored for 24 h at 25 °C. After 24 h, the number of dead larvae in each well was recorded. Death was induced on the remaining alive larvae by adding potassium dichromate (K_2_Cr_2_O_7_) at 1 mg/mL, and incubation occurred under the aforementioned conditions. After 24 h, all dead larvae were counted, and the mortality rate (%) was determined. DMSO at the same concentration of the samples was used as a negative control. Four replicates were used for each test, and the assay was performed in triplicate.

### 2.12. Statistical Analysis

The results were expressed as the mean value ± SD. The Shapiro–Wilk test was used for verification of the normality of the data. The Kruskal–Wallis with multiple comparisons of mean ranks and the one-way analysis of variance (ANOVA) with the Tukey post hoc test were used to determine differences between samples. The results were analyzed using STATISTICA 13.3 software (StatSoft, Tulsa, OK, USA). A probability level *p* < 0.05 was considered to indicate statistical significance.

## 3. Results

### 3.1. Hairy Roots Induction

**Figure 2:**
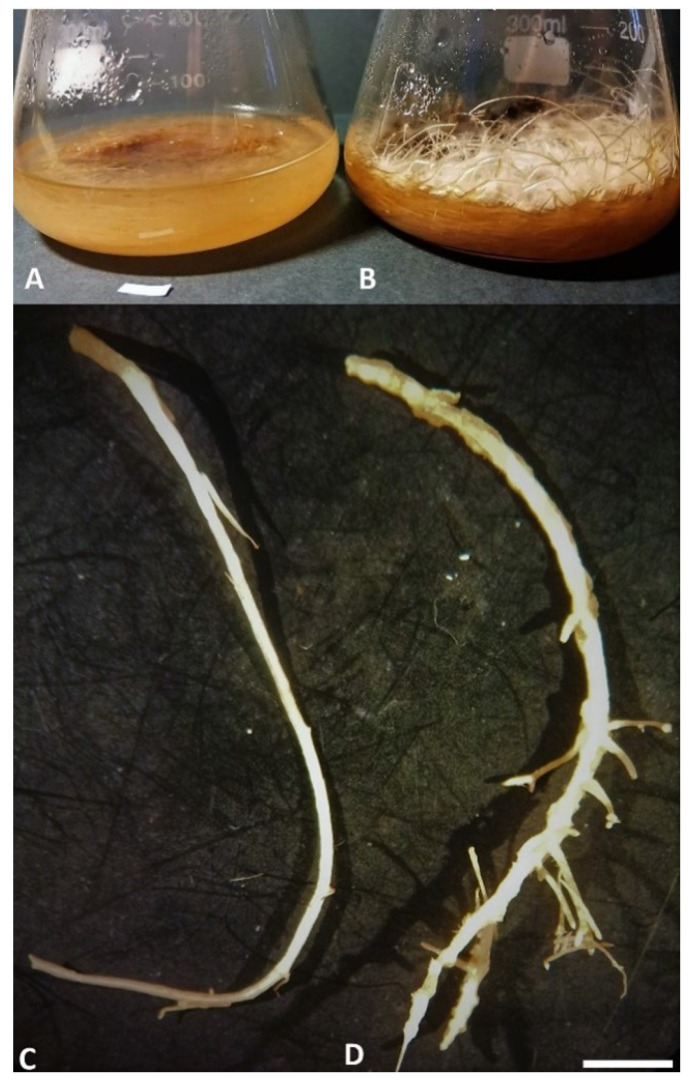
(**A**) Untransformed roots growing in a liquid medium, (**B**) hairy roots growing in a liquid medium, (**C**,**D**) differences in the structure of nontransformed (**C**) and hairy roots (**D**) of *L. nepetifolia* growing in vitro. Bar = 1 cm.

We observed the appearance of hairy roots only in the places where the plant material was injured and inoculated with the bacterial suspension, regardless of the applied method, after 14 days (Figure 1C). Well-developed transformed roots were observed on plant tissue 21 days after transformation (Figure 1D). Analysis of transformed and nontransformed root lines revealed differences in their structure (Figure 2C,D). Untransformed roots were thinner, showing a slower growth rate (80 ± 1.27 g/L of FW and 9.85 ± 0.35 g/L of DW from 2 g of inoculum) and fewer branches, while transformed roots grew faster (130.75 ± 4.3 g/L of FW and 16.3 ± 0.3 g/L DW from 2 g of inoculum), showing strong branching (Figure 2A,B). Higher transformation efficiency was obtained for methods using acetosyringone. The highest efficiency of hairy roots formation was obtained for the DM combined with acetosyringone (84% of seedlings revealed induction of hairy roots). Our results showed that using acetosyringone combined with method DM allowed to increase the efficiency of transformation, resulting in the induction of hairy roots (Table 1).

### 3.2. Confirmation of Transformed Nature of Hairy Roots

**Figure 3:**
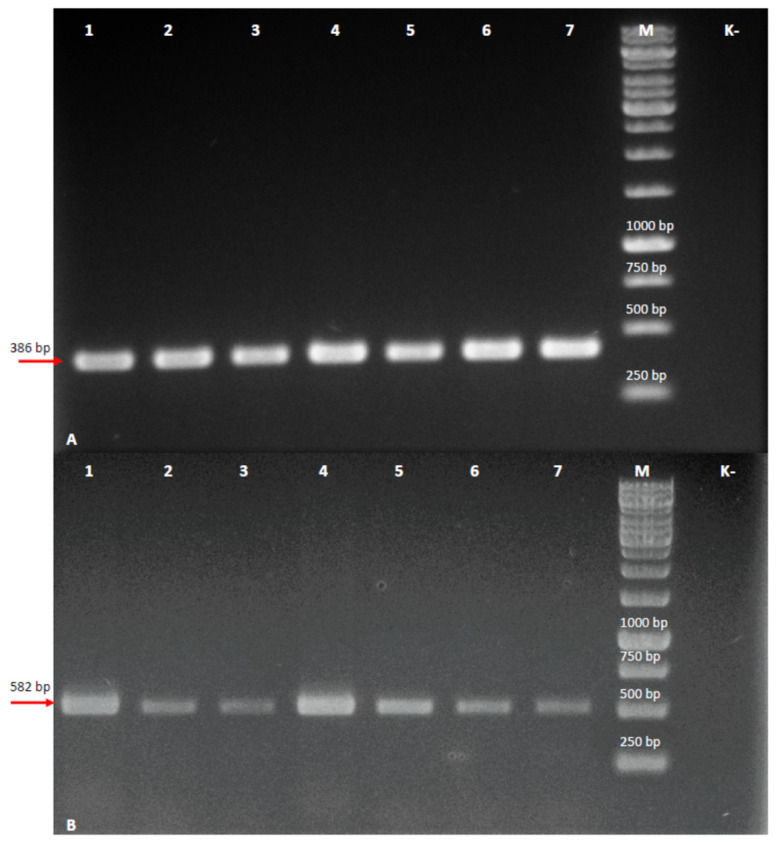
PCR detection of (**A**) *rol*B and (**B**) *rol*C genes in *L. nepetifolia*-independent hairy root clones. Lanes 1–7 = PCR on DNA isolated from hairy root lines; M = 1 kb DNA ladder, K- = PCR on DNA isolated from nontransformed root line.

PCR results confirmed the *rol*B and *rol*C genes in the genome of the obtained roots and thus their transgenic nature. Both for the *rol*B and the *rol*C genes, amplicons of the expected size (386 bp and 582 bp, respectively) were obtained, while they were not detected in DNA isolated from nontransformed roots (Figure 3).

### 3.3. Phytochemical Analysis of Two Extracts of L. nepetifolia

**Figure 4:**
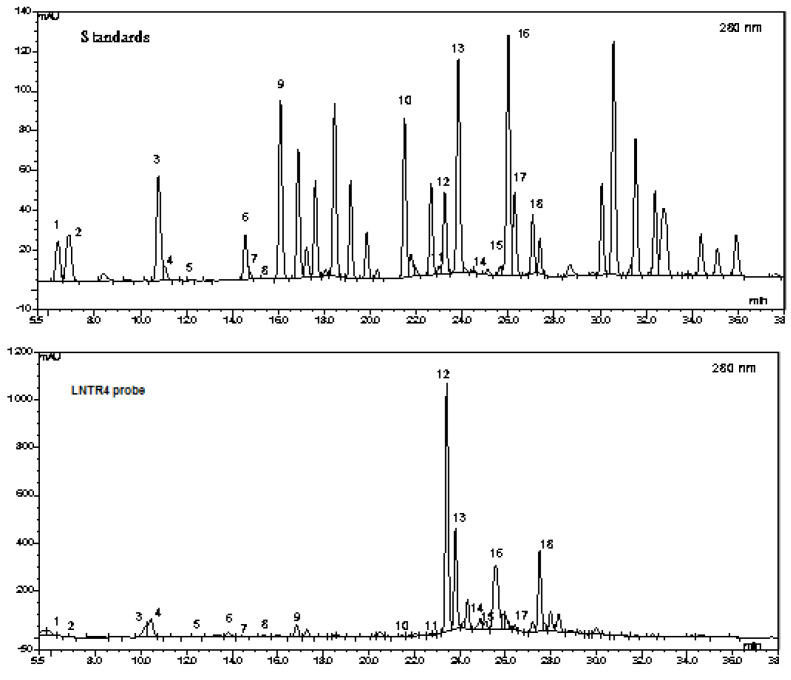
Representative HPLC chromatograms of a mixture of standard compounds and LNTR4 root extract of *L. nepetifolia*.

The polyphenols contained in the LNNR and LNTR4 roots extracts obtained from *Leonotis nepetifolia* by using an HPLC-MS method were identified: hydroquinone (1), gallic acid (2), *α*-resorcylic acid (3), catechol (4), protocatechuic acid (5), (+)-catechin (6), 4-hydroxybenzoic acid (7), gentisic acid (8), chlorogenic acid (9), *p*-coumaric acid (10), sinapic acid (11), coumarin (12), *m*-coumaric acid (13), rutin (14), ellagic acid (15), hesperidin (16) *o*-coumaric acid (17) rosmarinic acid (18). A typical chromatogram of LNTR4 root extract of *L. nepetifolia* is shown in Figure 4.

Our preliminary HPLC analyses showed different content of phenols and flavonoids in the tested extracts but a higher total amount of phenols and flavonoids in LNTR4 than in LNNR root extracts (21,838.95 and 15,913.84 µg/g DW, respectively) were demonstrated. In both tested extracts, the most dominant compounds were (+)-catechin (5464 and 6808 µg/g DW), *p*-coumaric acid (2549 and 4907 µg/g DW), *m*-coumaric acid (1508 and 2048 µg/g DW) and rosmarinic acid (1844 and 2643 µg/g DW) for LNNR and LNTR4 root extracts, respectively. All results are compiled in Table 2.

### 3.4. The Effect of LNNR and LNTR4 Root Extract from L. nepetifolia on Cell Viability

**Figure 5:**
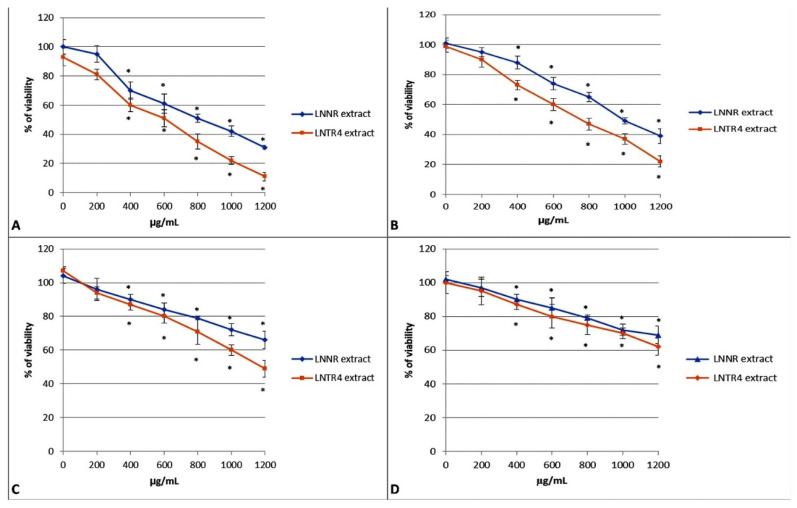
Cytotoxic effect of LNNR and LNTR4 root extracts on the viability of NALM-6 (**A**), HCC1937 (**B**), A549 cancer cell lines (**C**) and normal HUVEC cell line (**D**). Cell viability was determined based on dose–response curves obtained in the MTT assay. To compare the sensitivity of cells to LNNR and LNTR4 root extracts, data for each cell line were presented at concentrations ranging from 200 to 1200 µg/mL after incubation for 24 h. All experiments were performed in triplicate, and results are expressed as mean ± SD. * *p* < 0.05 comparison untreated cells vs. LNNR, LNTR4 extract.

The effects of LNNR and LNTR4 root extracts from *L. nepetifolia* were analyzed after 24 h incubation with the extracts at concentrations in the range of 200–1200 µg/mL in human lung adenocarcinoma A549 cell line, breast cancer cell line HCC1937, NALM-6 leukemia cancer cell line and human umbilical vein endothelial cells HUVEC cell line. The viability of NALM-6 and HCC1937 cancer cells was significantly inhibited by both tested extracts with IC_50_ = 900 µg/mL for LNNR root extract and IC_50_ = 550 µg/mL for LNTR4 root extract, respectively for NALM-6 cells, and with IC_50_ = 1000 µg/mL for LNNR root extract and IC_50_ = 750 µg/mL for LNTR4 root extract, respectively for HCC1937 cells, but a stronger activity for LNTR4 root extract was observed **(**Figure 5A,B). Moreover, for LNTR4, the root extract was IC_50_ = 1200 µg/mL, but for LNNR root extract, the IC_50_ was not reached in the tested concentration range for the A549 lung cell line (Figure 5C). There was only a slight decrease in viability of the HUVEC normal cells after both extracts at 24 h in the concentration range tested (Figure 5D). In addition, we did not see a decrease in survival HUVEC cells after treatment with 75 or 100 µM H_2_O_2_ (data not shown).

### 3.5. Effect of LNNR and LNTR4 Root Extracts on ROS Level

**Figure 6:**
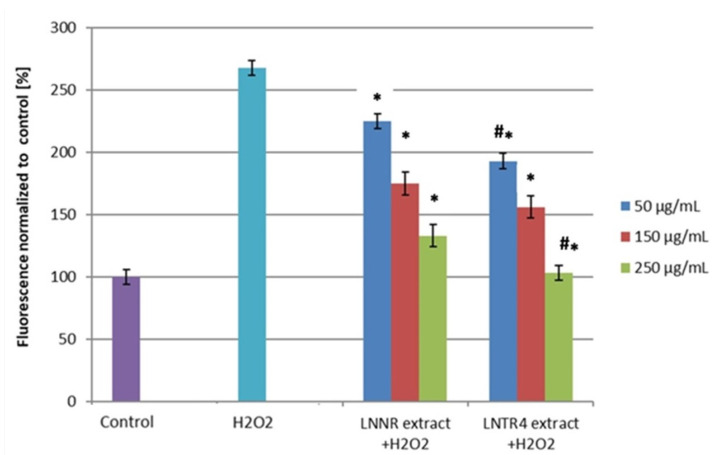
Effect of LNNR and LNTR4 root extracts of *L. nepetifolia* on H_2_O_2_-induced reactive oxygen species (ROS) generation in HUVECs cells. The cells were pretreated (for 24 h) with different concentrations of both extracts before exposure (1 h) to 100 µM of H_2_O_2_. Data are expressed as mean ± SD of three independent experiments. * *p* < 0.05 comparison control vs. LNNR, LNTR4 extracts, # *p* < 0.05 comparison LNNR vs. LNTR4 extracts.

The production of ROS was determined following H_2_O_2_ exposure of LNRR and LNTR4 root extracts of *L. nepetifolia* treatment. As we expected, hydrogen peroxide (H_2_O_2_) caused a significant increase in ROS level in HUVEC cells compared to the control. Both tested extracts, at concentrations of 50, 150, and 250 µg/mL, decreased the intracellular ROS level, but the stronger effect of LNTR4 root extract was observed, and the reduction in ROS expression increased with the tested extract concentrations (Figure 6). At a concentration of 150 µg/mL, both extracts did not reduce ROS significantly.

### 3.6. Antioxidant Activity (DPPH Assay)

**Figure 7:**
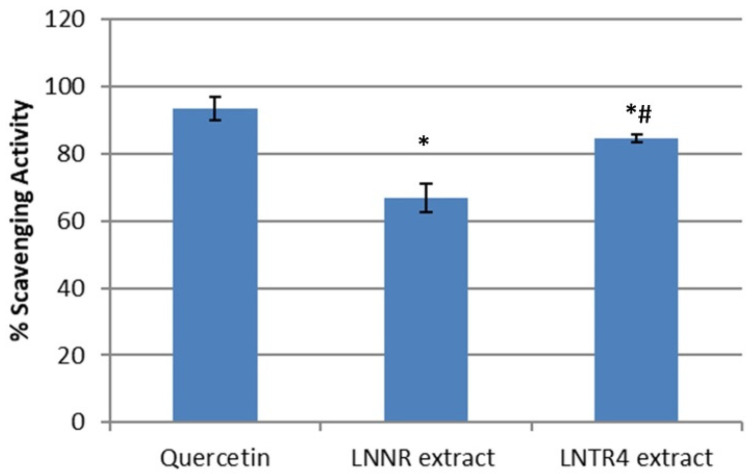
Antioxidant activity of LNNR and LNTR4 root extracts of *L. nepetifolia* under study tested at a concentration of 10 µg/mL. Values are expressed as the mean ± SD (*n* = 3). * *p* < 0.05 comparison control vs. LNNR and LNTR4 extracts, # *p* < 0.05 comparison LNNR vs. LNTR4 extracts.

The antioxidant activities of the LNNR and LNTR4 root extracts in this study were evaluated using the DPPH method. Both tested extracts showed the higher scavenging activity (86% and 70%) for LNTR4 and LNNR, respectively, compared to the control, but the LNTR4 root extract showed the stronger antioxidant effect (Figure 7).

### 3.7. DNA Repair after Treatment of LNNR and LNTR4 Root Extracts from L. nepetifolia

**Figure 8:**
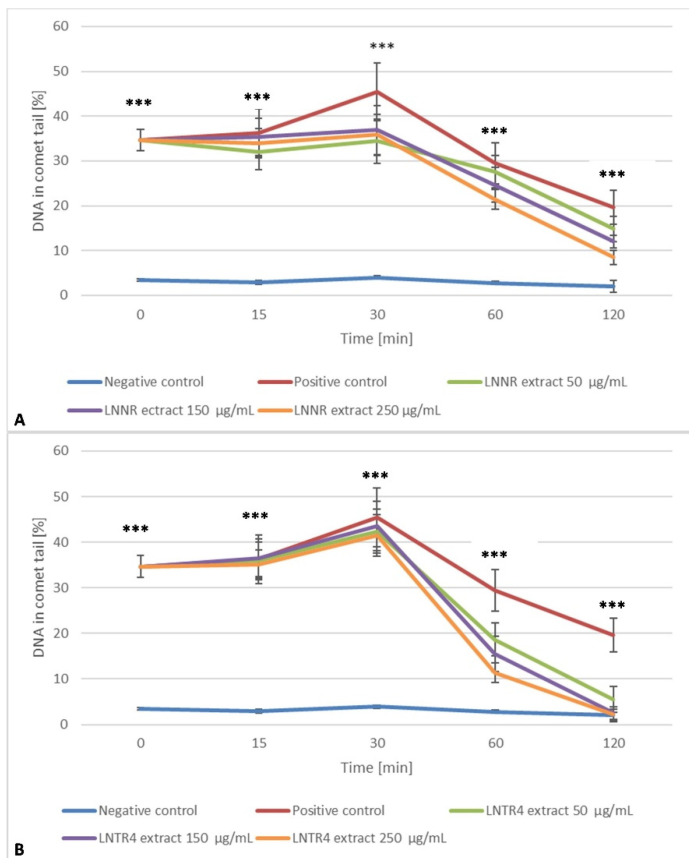
LNNR (**A**) and LNTR4 (**B**) root extracts showed DNA damage in human umbilical vein endothelial cells (HUVEC), treated with 75 μM hydrogen peroxide for 10 min at 4 °C (time 0), then incubated with both extracts within the range of concentrations from 50 to 250 µg/mL and measured for 15, 30, 60, and 120 min. Negative control is the lack of damage agents. Positive controls were treated with hydrogen peroxide alone. The values were measured as the mean percentage of DNA in the comet’s tail ± SD in the alkaline version of the comet assay. The tail DNA fraction was determined for 50 comets in each trial. *** *p* < 0.001 comparison control vs. extracts with H_2_O_2_.

Our study showed that, at time 0, the level of DNA damage in HUVEC cells generated by hydrogen peroxide was statistically significantly higher (*p* < 0.001) compared to the negative control (without the damaging factor). DNA damage was completely repaired by cells during incubation with all the concentrations (50, 150 and 250 µg/mL) of LNTR4 root extract, as well as 250 µg/mL of LNNR root extract during the 120 min repair incubation (*p* > 0.05). The cells exposed to hydrogen peroxide and incubated with LNNR root extract with 50 µg/mL (*p* = 0.003) and 125 µg/mL (*p* < 0.001) were not able to effectively repair DNA damage in 120 min. It was observed that cells exposed to LNTR4 root extract more efficiently repaired the oxidative DNA damage induced by hydrogen peroxide. The efficient repair capability within the whole range of analyzed concentrations may be related to an elevated level of polyphenolics content in LNTR4 root extract compared to an LNNR root extract (Figure 8A,B).

### 3.8. DNA Protective Effect after Pretreatment with LNNR and LNTR4 Root Extracts

**Figure 9:**
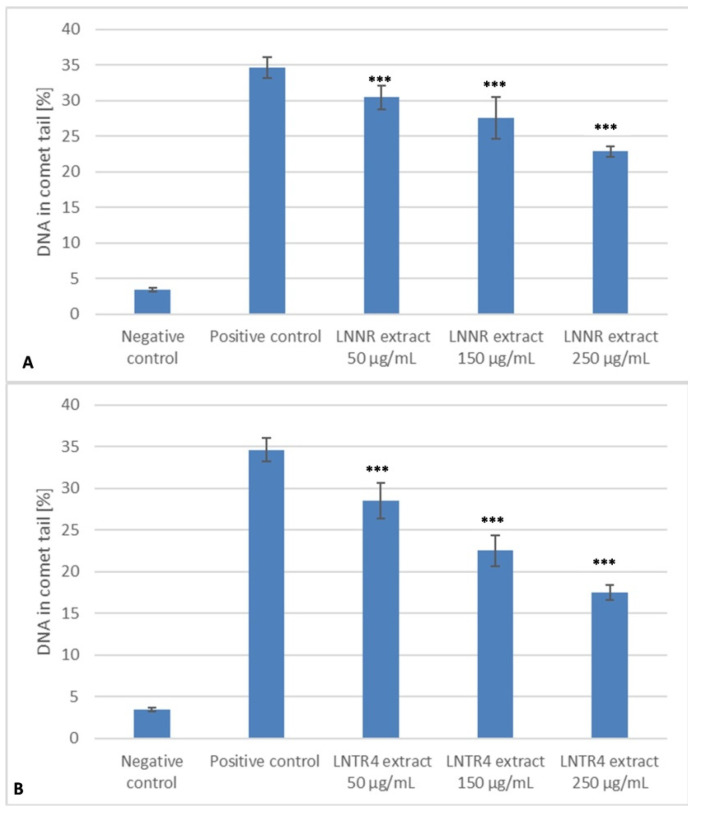
LNNR (**A**) and LNTR4 (**B**) root extracts show DNA’s protective effect in human umbilical vein endothelial cells (HUVECs) pretreated with both extracts within the range of concentrations from 50 to 250 µg/mL for 24 h and 75 μM hydrogen peroxide for 10 min. Negative control is the lack of damage agents. Positive controls were treated with hydrogen peroxide alone. The values were measured as the mean percentage of DNA in the comet’s tail ± SD in the alkaline version of the comet assay. The tail DNA fraction was determined for 50 comets in each trial. *** *p* < 0.001 comparison control vs. extracts with H_2_O_2_.

Both tested extracts were analyzed for their protective ability against the oxidative DNA damage generated in human lymphocytes by hydrogen peroxide—positive control. As shown in Figure 9A,B, the pretreatment of cells with both tested extracts within the range of concentrations from 50 to 250 µg/mL for 24 h at 37 °C resulted in the different degree of DNA protection from oxidative-induced damage. When used at 250 µg/mL concentration, the plant extracts showed much higher protection than at other concentrations. Additionally, LNTR4 root extract DNA that had been protected was more effective than LNNR root extract. The DNA damage after hydrogen peroxide treatment, measured as a percentage of DNA in the comet’s tail ± SD, had a value of 34.65 (±2.43). The extract pretreatment significantly reduced DNA damage than the positive control to 17.52% (±2.49) and 22.84% (±2.49) in 250 µg/mL doses for the LNTR4 and the LNNR, respectively. The differentiation of extract protection ability against oxidative-induced DNA damage, and specifically LNTR4 root extract efficient protection, may be related to higher polyphenolics content.

### 3.9. Brine Shrimp Lethality Bioassay after Treatment of LNNR and LNTR4 Root Extracts

**Figure 10:**
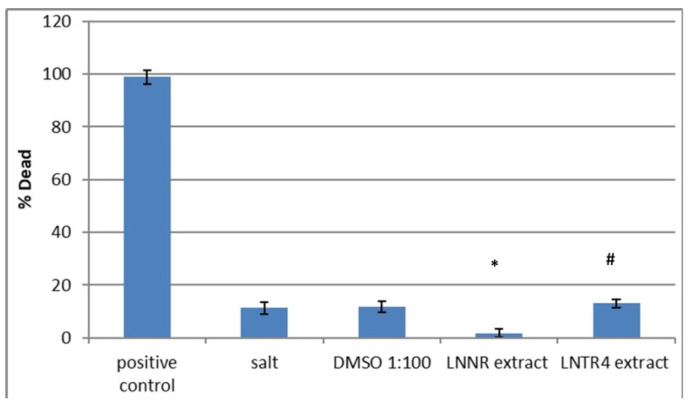
Mortality rate (%) of *Artemia salina* after 24 h exposure to LNNR and LNTR4 root extracts from *L. nepetifolia.* Values are expressed as the mean ± SD (*n* = 3). * *p* < 0.05 comparison salt vs. LNNR and LNTR4 extracts, # *p* < 0.05 comparison LNNR vs. LNTR4 extracts.

The brine shrimp lethality bioassay was used to predict the cytotoxic activity of the LNNR and LNTR4 root extracts from *L. nepetifolia*. The percentage mortality rate of the brine shrimp lethality bioassay obtained for these extracts and the positive control are shown in Figure 10. Both tested extracts exhibited significant toxicity towards brine shrimps. The mortality rates of the plant root extracts (500 µg/mL) were about 12% for LNTR4 root extract, that is, at the level of the negative control with salt (12%) and about 2% for LNNR root extract, whereas that of the positive control was about 98%. Therefore, our preliminary in vivo studies have shown that the tested extracts do not have a toxic effect on brine shrimp in the tested concentration range, so they can be used for further studies on a different model.

## 4. Discussion

Plant cell culture techniques developed in the past as possible tools for producing secondary metabolites are still used with great success. Hairy roots have been the research focus for many years due to the simple, effective and cheap breeding, rapid growth, increased production of metabolites and better biological properties than many species of plants found in nature [2,28]. This fast growth, genetic and biochemical stability, ease of maintenance and ability to synthesize various biologically active compounds offer an additional advantage over other undifferentiated plant cell cultures. Furthermore, transformed hairy roots can synthesize more than one metabolite simultaneously. It is one of the most productive models in green biotechnology for obtaining new bioactive compounds. Therefore, it has become a valuable production method even on an industrial scale [5].

In the present study, for the first time, we have successfully obtained *Leonotis nepetifolia* transformed roots by *Rhizobium rhizogenes-*mediated transformation. In addition, our preliminary studies showed different phenol and flavonoids content in transformed roots compared to the non–transformed roots, and we also demonstrated different biological properties of the obtained extracts.

Many methods for genetic modification of plants are reported in the literature. However, the most common and effective strategy is transformation by *Rhizobium rhizogenes*, also known as “natural genetic engineer”, and inducing characteristic roots at the site of infection, resulting from introducing bacterial genetic material (T-DNA) into the plant genome [29]. Our previous studies have successfully established and confirmed the transformed nature of hairy roots from different plant species by the presence of *rol*B and *rol*C genes in their genomes [10,30]. It is known that the mechanism of plant genetic transformation depends on many factors, including mainly the activity of *R. rhizogenes* induced by phenolic compounds released from the sites of plant tissue injury, which in turn induce bacterial *vir* genes. One of the commonly used inducers influencing the transformation efficiency is acetosyringone [31]. Our research has shown increased the effectiveness of hairy root induction in transformation procedures with acetosyringone (84% and 62%) for the DM and IM method, respectively, compared to the effectiveness of hairy root induction without this compound (50% and 34%). These results are consistent with Brijwal et al., who showed that acetosyringone also caused a higher frequency of *Berberis aristata* hairy roots induction [32]. Similarly, Saleh et al. showed that acetosyringone enhanced forming hairy roots in *Solenostemon scutellarioides* [33].

Our preliminary phytochemical analysis has shown differences in the content of polyphenols in transformed roots of *Leonotis nepetifolia* compared to the nontransformed sample. Literature data revealed that the transformation of *Rhizobium rhizogenes* could influence the increased production of bioactive compounds by changing gene expression patterns in selected metabolic pathways [2,34]. Our previous studies showed that transformed *Leonurus sibiricus* roots produced higher amounts of phenolic acids than nontransformed roots [10]. El-Esawi et al. noted that transformed roots of *Lactuca serriola* enhanced the production of phenolics and flavonoids [35]. Similar conclusions were also reached by Huang et al., who showed that the transformed roots of *Gentiana scabra* produced increased amounts of iridoid and secoiridoids [36]. Our results are consistent and showed an increased content of phenols and flavonoids in the transformed roots of *L. nepetifolia*. To the author’s knowledge, this is the first report on the preliminary phytochemical analysis of transformed and nontransformed roots of *L. nepetifolia*, where it was shown that the dominant compounds were: (+)-catechin (5464 and 6808 µg/g DW), *p*-coumaric acid (2549 and 4907 µg/g DW), *m*-coumaric acid (1508 and 2048 µg/g DW) and rosmarinic acid (1844 and 2643 µg/g DW), respectively.

The preliminary cytotoxic screening revealed that both root extracts of *L. nepetifolia* reduced the survival rate of leukemia NALM-6 and breast HCC1937 cancer cells in the concentration range tested (200–1200 µg/mL), but only for LNTR4 extract for A549 lung cancer cells. The NALM-6 leukemia cell line was the most sensitive to the effects of LNTR4-transformed root extract with IC_50_ = 550 µg/mL. We suspect that this stronger effect may be responsible for the higher phenols and flavonoids content in the LNTR4 root extract (especially (+)-catechin, *p*-coumaric acid, *m*-coumaric acid or Rosmarinic acid). This is the first study of in vitro *L. nepetifolia* root extracts. Our previous studies revealed that transformed root extract from *Leonurus sibiricus,* where the most dominant compounds were the phenolic acids, possessed a cytotoxic effect on two leukemia cell lines (K542 and CCRF-CEM) [37]. In another study, Veerabadran et al. showed that extracts from *L. nepetifolia* leaves have a toxic effect on Hep2 liver and MCF-7 breast cancer cells with IC_50_ = 1250 µg/mL. The authors hypothesize that phenols and flavonoids contained in the extracts may be responsible for these properties [38]. In turn, Sobolewska et al. demonstrated that extracts from aerial parts of this plant exhibited cytotoxic in vitro activity towards Du145 human prostate cancer cell line with IC_50_ = 100 and 60 µg/mL [14]. According to the literature, polyphenols act on carcinogenesis through the induction of cell defense systems, including detoxifying and antioxidant enzyme systems, as well as the inhibition of the anti-inflammatory and anti-cellular growth signaling pathways that culminate in cell cycle arrest and/or cellular death. These contributions strongly suggest the anti-cancer effects of polyphenols due to their ability to alter the epigenome of cancer cells [39,40,41]. Additionally, despite the observed activity against tumor cell lines, both tested LNNR and LNTR4 root extracts of *L. nepetifolia* exhibited no toxicity against brine shrimp, which indicated their potential safety for living organisms in the tested concentration. Miceli et al. presented cytotoxic *Brassica incacana* extracts’ efficacy against Caco-2 cells (about 90% activity at the highest concentration tested), but in the brine shrimp lethality bioassay, the extracts exhibited no toxicity, indicating their potential safety [42]. Similarly, Savkin et al. demonstrated that *Cornus mas* and *Cotinus coggygria* extracts possessed potential cytotoxic activity towards HeLa and LS174 human cancer cell lines in vitro, but there were no significant changes in toxicity in the brine shrimp lethality test [43].

Plant-based antioxidants protect biological systems against oxidative stress generated by free radicals or reactive oxygen species (ROS) during metabolism and other activities. Free radicals are known to play a role in a wide variety of pathological manifestations. Antioxidants fight free radicals and protect from various diseases (Parkinsonism, hypertension, ischemic diseases, atherosclerosis, Alzheimer’s disease or cancer). They exert their effects by scavenging reactive oxygen species or by protecting antioxidant defense mechanisms [44,45]. Our results showed a strong antioxidant effect in the DPPH radical scavenging ability of LNNR (70%) and LNTR4 (86%) root extracts of *L. nepetifolia*. Veerabadran et al. confirmed the antioxidant properties of leaf extracts using the DPPH test [38]. In both cases, the phenols and flavonoids contained in the extracts were the main compounds. On the other hand, Sobolewska et al. noted that extracts from aerial parts of *L. nepetifolia* had low total antioxidative ability determined using FRAP and DPPH methods. The authors speculated that this effect might be related to the low phenolic content in the tested extracts [14].

Our study, confirming for the first time the antioxidant properties of the LNNR and LNTR4 root extracts of *L. nepetifolia,* was carried out on an in vitro model and showed a reduction of ROS level in HUVEC cells induced with hydrogen peroxide (H_2_O_2_). Both extracts showed such properties, but a stronger effect was observed for LNTTRAs suggested above. This effect may be related to the higher content of phenols and flavonoids. These results indicate that the LNNR and LNTR4 root extracts show the potential capacity to decrease ROS production in HUVECs under oxidative stress conditions. Our research is consistent with Jia et al., who showed that flavonoids from *Rosa laevigata* Michx fruit extract decreased ROS levels in H_2_O_2_-induced HUVEC cells [46].

Additionally, in this study, we also investigated the protective and repairing effect of HUVEC cells on DNA damage induced by H_2_O_2_. We revealed that both extracts could stimulate the repair mechanism in the HUVEC cells induced by H_2_O_2_ and protect them from DNA damage induced by this compound in a dose-dependent manner. Our previous studies showed that in vitro extracts from *Leonurus sibiricus* rich in phenolic compounds stimulated repair and protective activity against oxidative DNA damage in CHO cells [23]. In turn, Behravan et al. demonstrated that the extract of *Portulaca oleracea* rich in flavonoids could prevent H_2_O_2_-induced DNA damage in lymphocytes [47]. Shojaee et al. reported that *Scutellaria litwinowii* root extract with a high amount of phenolic and flavonoid compounds could have a protective effect against DNA damage caused by H_2_O_2_ scavenging of free radicals in NIH 3T3 cells [48]. A similar effect was achieved by Cheng et al. after treatment of honey extracts rich in phenolic compounds, which, by penetrating into lymphocytes, can protect DNA against oxidative damage by scavenging H_2_O_2_ [49]. In all the examples presented, the phenols and flavonoids contained in the tested extracts were responsible for the protective effect. In our research, we also suppose that these compounds may present such properties, more so that our phytochemical analysis confirmed their presence in the extracts with various amounts, and even though both extracts showed a protective effect, the LNTR4 root extract showed it had the stronger properties. The main compound in this extract was (+)-catechin, which, according to the literature, is one of the stronger antioxidants [50,51].

## 5. Conclusions

The data from this study illustrated that LNTR4 transformed root extract of *L. nepetifolia,* containing a higher amount of phenols and flavonoids, revealed a stronger antioxidant and cytotoxic effect. Therefore, LNTR4 root extract could provide stronger activity in repairing and protecting HUVEC cells against H_2_O_2_-induced DNA damage than LNNR nontransformed root extract. These effects, we surmise, are likely due to phenolic constituents that are acting as antioxidants. One of the putative mechanisms for this observed effect may be free radical scavengers. However, more in-depth studies of this mechanism and in vivo experiments are needed before any conclusions can be drawn about the greater use of these extracts for human health.

## Figures and Tables

**Table 1:** Efficiency of transformation of *L. nepetifolia* with the use of *R. rhizogenes* depending on the method used.

Acetosyringone			No Acetosyringone		
No. of Seedlings Per Treatment	No. of Seedlings With Hairy Roots	Hairy Roots Induction Efficiency (%)	No. of Seedlings Per Treatment	No. of Seedlings with Hairy Roots	Hairy Roots Induction Efficiency (%)
DM method					
50	42	84	50	28	50
IM method					
50	31	62	50	17	34

**Table 2:** Contents of phenolic compounds in extracts from normal (LNNR) and transformed (LNTR4) roots of *L. nepetifolia*.

	Compounds	LNNR Extract µg/g DW	LNTR4 Extract µg/g DW
1	Hydroquinone	126.9 ± 0.298 ^b^	107.7 ± 0.369 ^a^
2	Gallic acid	115.14 ± 0.994 ^a^	117.78 ± 0.825 ^a^
3	*α*-Resorcylic acid	168.52 ± 3.034 ^a^	161.62 ± 0.366 ^a^
4	Catechol	731.62 ± 0.192 ^a^	714.2 ± 1.854 ^a^
5	Protocatechuic acid	722.36 ± 0.834 ^b^	657.28 ± 0.259 ^a^
6	(+)-Catechin	5464 ± 13.31 ^a^	6808 ± 10.64 ^b^
7	4-Hydroxybenzoic acid	325.4 ± 0.532 ^b^	209.2 ± 0.426 ^a^
8	Gentisic acid	118.44 ± 0.02 ^a^	118.78 ± 0.831 ^a^
9	Chlorogenic acid	152.22 ± 1.044 ^a^	264.6 ± 0.410 ^b^
10	*p*-Coumaric acid	2549 ± 3.393 ^a^	4907 ± 13.73 ^b^
11	Sinapic acid	2.382 ± 0.031 ^a^	14.39 ± 0.223 ^b^
12	Coumarin	981.6 ± 1.692 ^a^	1472.4 ± 2.563 ^b^
13	*m*-Coumaric acid	1508.5 ± 4.452 ^a^	2048 ± 2.759 ^b^
14	Rutin	230.9 ± 3.653 ^a^	261.6 ± 0.185 ^b^
15	Ellagic acid	232.00 ± 0.435 ^a^	455.4 ± 0.576 ^b^
16	Hesperidin	442.00 ± 1.192 ^a^	603.8 ± 0.419 ^b^
17	*o*-Coumaric acid	198.86 ± 0.673 ^a^	274.2 ± 0.934 ^b^
18	Rosmarinic acid	1844 ± 3.407 ^a^	2643 ± 2.401 ^b^
	Total sum of phenols and flavonoids	15,913.842 ± 39.18 ^a^	21,838.95 ± 39.77 ^b^

Different superscript letters within the rows indicate significant differences in the mean values at *p* < 0.05.

## Data Availability

All data are included in the manuscript.
